# Endoscopic Optical Coherence Tomography for Clinical Gastroenterology

**DOI:** 10.3390/diagnostics4020057

**Published:** 2014-05-05

**Authors:** Tsung-Han Tsai, James G. Fujimoto, Hiroshi Mashimo

**Affiliations:** 1 Department of Electrical Engineering and Computer Science and Research Laboratory of Electronics, Massachusetts Institute of Technology, Cambridge, MA 02139, USA; E-Mails: johann.tsai@alum.mit.edu (T.-H.T.); jgfuji@mit.edu (J.G.F.); 2 Veterans Affairs Boston Healthcare System and Harvard Medical School, Boston, MA 02115, USA

**Keywords:** optical coherence tomography, optical biopsy, endoscopic imaging, Barrett’s esophagus, inflammatory bowel disease

## Abstract

Optical coherence tomography (OCT) is a real-time optical imaging technique that is similar in principle to ultrasonography, but employs light instead of sound waves and allows depth-resolved images with near-microscopic resolution. Endoscopic OCT allows the evaluation of broad-field and subsurface areas and can be used ancillary to standard endoscopy, narrow band imaging, chromoendoscopy, magnification endoscopy, and confocal endomicroscopy. This review article will provide an overview of the clinical utility of endoscopic OCT in the gastrointestinal tract and of recent achievements using state-of-the-art endoscopic 3D-OCT imaging systems.

## 1. Introduction

**Figure 1:**
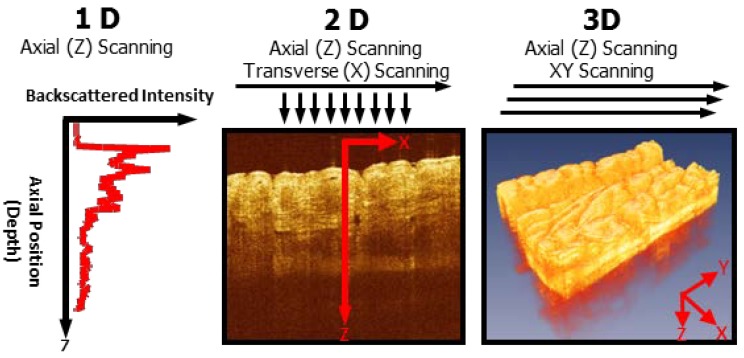
Optical coherence tomography (OCT) generates cross-sectional and 3D images of tissue microstructure by measuring the echo time delay and magnitude of backscattered light. Architectural morphology can be imaged *in vivo* and in real time.

Optical coherence tomography (OCT) is an imaging technology that enables micron scale, cross-sectional and three-dimensional (3D) imaging of sample microstructure in real time [1,2,3] and can work as an “optical biopsy” tool for biomedical applications; *i.e.*, microstructural information of tissue can be obtained *in vivo* with resolutions approaching that of histopathology on excisional biopsy without needing to remove tissue specimens or apply additional contrast agents on the tissue [4,5,6]. OCT can provide nondestructive profilometry as well as 3D analysis of depth-resolved features. OCT is an optical analog of ultrasound B mode imaging, which is performed by measuring the echo time delay and intensity of back-reflected or backscattered light. As shown in Figure 1, an optical beam is scanned across the sample and echoes of backscattered light are measured as a function of axial range (depth) and transverse position. 3D imaging is obtained by performing a two-dimensional scan pattern at different transverse positions. Three-dimensional OCT (3D-OCT) enables powerful methods for visualizing tissue architecture. 3D-OCT generates comprehensive, volumetric data sets, which can be used to construct arbitrary cross-sectional images, projections along arbitrary axes, or 3D renderings similar to magnetic resonance imaging (MRI) or computed tomography (CT).

**Figure 2:**
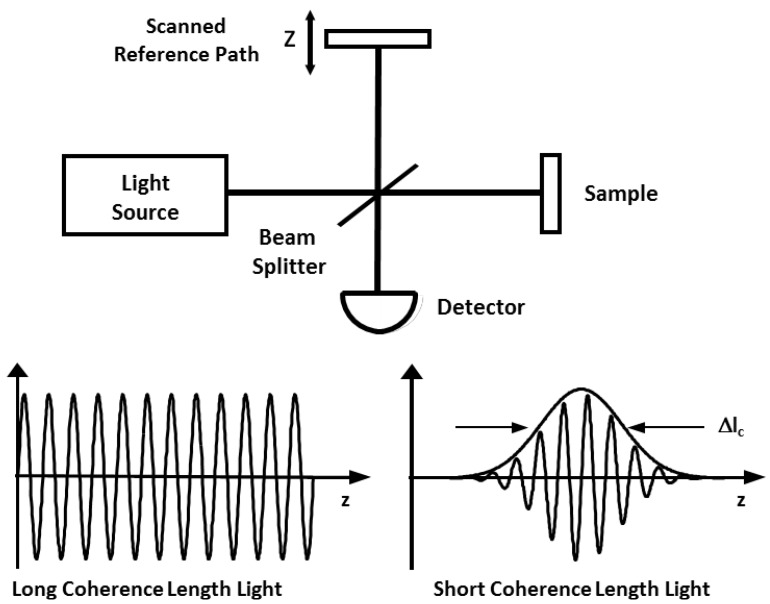
OCT uses low coherence interferometry to detect the time delay and magnitude of backscattered light.

OCT is based on a technique known as low coherence interferometry, which has been previously applied to perform optical ranging measurement [7,8,9]. OCT imaging is performed using a Michelson interferometer with a short coherence length light source as shown in Figure 2. One arm of the interferometer contains a scanning system that scans the light beam over the sample (XY scan shown in Figure 1) and collects the backscattered light. In most biomedical applications, this is usually a microscope for imaging excised tissue specimens or small animals *in vivo*, or an endoscopic fiber optic imaging catheter for imaging inside the body [6]. The scanning system determines the field of view (FOV) and the transverse resolution, which defines the scale and detail of *en face* features that the OCT system can reveal. The second arm of the interferometer has a scanning reference path delay that is mechanically translated over the desired imaging depth in the traditional time domain OCT (TD-OCT) configuration. Optical interference between the light from the sample and reference occurs only when the optical delays match to within the coherence length of the light [10,11]. “Coherence” refers to a temporal property of the light, which is inversely proportional to its wavelength bandwidth and quadratically proportional to its central wavelength. Low coherence interferometry enables the echo delay time and magnitude of backscattered light from internal tissue microstructures to be measured with high time resolution and sensitivity.

In the early 2000s, dramatic advances in OCT technology have resulted in imaging speeds 10–200 times faster and detection sensitivity 10–100 fold greater than standard TD-OCT by using a new OCT detection technique known as Fourier domain detection. Measuring backscattered light in the Fourier domain [12,13,14,15] has enabled real-time 3D-OCT imaging for *in vivo* biomedical applications. While conventional OCT directly measures the interference signal, Fourier domain OCT measures the spectrum of the interference signal. The OCT axial scan is then obtained by applying Fourier transformation on the detected spectrum [13,14,15]. This Fourier domain OCT includes two different types of techniques, namely spectral/Fourier domain OCT (SD-OCT) and swept source OCT (SS-OCT, also known as optical Fourier domain imaging, OFDI). Spectral/Fourier domain detection uses a spectrometer with a high speed, line scan camera to measure the spectrum of the OCT interference signal. SD-OCT typically operates at 800 nm or 1 µm wavelengths with imaging speed of 29,000–300,000 lines per second [16,17,18,19,20]. This technology has had a great impact especially on ophthalmic OCT imaging because it enables ultrahigh resolutions as well as 3D imaging of retinal pathologies *in vivo* for the first time [21,22].

**Figure 3:**
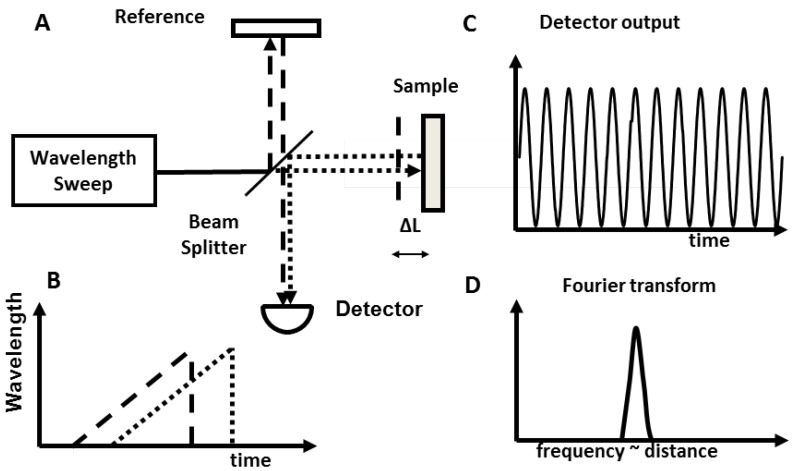
Swept source OCT enables a 10–200× increase in imaging speed compared to time domain OCT. (**A**) Interferometer with path difference ΔL and swept laser light source. (**B**) Light from the sample (dotted) and reference path (dashed) are time delayed. (**C**) Interference beat signal frequency is proportional to delay. (**D**) Fourier transform of beat signal measures the delay ΔL.

On the other hand, SS-OCT uses a wavelength-swept laser light source and a photodetector to measure the interference spectrum [23,24,25,26], as shown in Figure 3. SS-OCT enables operation at longer wavelengths of 1 µm and 1.3 µm and does not need expensive indium gallium arsenide (InGaAs) cameras. Imaging at this wavelength range reduces optical scattering and improves imaging penetration depths in the tissues [5]. Advances in wavelength-swept lasers have enabled multi-megaherz high speed OCT imaging [27,28,29,30,31]. This technology is well suited for imaging scattering tissues such as in the colon, esophagus, and breast ducts, since scattering significantly increases at shorter wavelengths.

Compared to SD-OCT, SS-OCT can have improved sensitivity because there is no spectrometer loss and photodiodes used for detection are more sensitive than cameras [32,33,34], so structural features with lower reflectivity can be revealed from the background of OCT images. SS-OCT can also achieve improved system dynamic range because it uses high speed and low-noise photo-detectors and higher bit depth data acquisition systems, so more detailed structural features corresponding to different reflectivity can be distinguished in the OCT images. Moreover, swept source/Fourier domain detection can provide a very large number of axial samples, as determined by the speed of the data acquisition systems. More axial samples enable an OCT signal with a higher interference beat signal frequency to be acquired correctly, corresponding to the information from the deeper regions. Currently, SS-OCT is especially well suited for applications requiring the fastest possible imaging speeds at wavelengths of 1.3 µm and 1 µm. On the other hand, SD-OCT provides a crucial resolution advantage (<3 µm) using light source with broader selection of operation wavelength and bandwidth, as the axial resolution of OCT is quadratically proportional to the center wavelength of the light source [16,35,36]. Therefore, for applications where slower speeds are acceptable, but axial resolution is of critical importance, such as retinal imaging, SD-OCT remains the dominant technology.

## 2. Utility of Endoscopic OCT in the Gastrointestinal Tract

Gastrointestinal (GI) cancers are estimated to account for more than 290,000 new cases of cancers and cause more than 140,000 deaths in the United States in 2014 [37], but are difficult to detect early enough to impact on mortality owing to their insidious onset. They often arise in the setting of chronic inflammation or injury, and early diagnosis currently depends largely on endoscopic evaluation and procurement of tissues for pathological confirmation.

### 2.1. Detection of Diseases in the GI Tract

Esophageal cancer has a five-year survival rate of only 16% [38]. In the past two decades, the incidence of esophageal squamous carcinoma has declined in the United States, but the incidence of esophageal adenocarcinoma bears the notoriety of having the most rapid rise of all cancers. Barrett’s esophagus (BE) is believed to arise from chronic mucosal injury caused by gastroesophageal reflux disorder and to be a precursor of esophageal adenocarcinoma [39]. BE is characterized by the replacement of squamous epithelium with columnar epithelium in the esophagus caused by chronic exposure to the acid environment [40]. Neoplastic changes in BE are thought to develop in stages from non-dysplastic metaplasia to increasing grades of dysplasia and eventually to adenocarcinoma [41]. Thus, early detection and ablation of the preceding dysplasias is a promising strategy for reducing the risk of adenocarcinoma [42].

Colorectal cancer is another common GI disease with high morbidity and mortality rates. Colorectal cancer is the third leading cause of cancer death in the United States, accounting for about 10% of cancer death overall [38]. Despite its high incidence, colorectal cancer is one of the most detectable, and, if found early, most treatable forms of cancer. Although most colorectal cancers arise from adenomatous polyps that are detectable using conventional endoscopy, many flat (non-polypoid) lesions are missed during routine examinations [43]. Up to 50% of these more subtle lesions are missed by conventional endoscopy [44]. This is particularly relevant in patients with inflammatory bowel diseases (IBDs) such as ulcerative colitis (UC) and Crohn’s disease (CD), where neoplastic tissue can be flat rather than polypoid in form and multifocal in distribution thereby evading endoscopic detection [45]. As many as 1.4 million individuals in the United States have IBDs [46] and are at increased risk for the development of colorectal cancer [47].

The current standard procedure to detect dysplasia or early stage cancer is to perform biopsy in the identifiably abnormal regions using endoscopy, or random biopsies over a broad area (such as entire length of BE or entire length of colon in patients with history of pancolitis) and diagnosis is made according to the histology results [48,49]. Besides inadequate sampling, the problem of early detection of dysplasias in the gut is exacerbated in the presence of chronic inflammatory conditions such as esophagitis and IBD, since early-stage lesions are difficult to distinguish from inflamed GI mucosa by the endoscopist. Thus, an imaging modality such as OCT which can provide broad-field, sub-surface, and near-microscopic imaging capabilities during endoscopy could help differentiate neoplastic tissue from inflamed tissue and help target areas for biopsy or ablation.

### 2.2. Assessment of Endoscopic Therapies for GI Diseases

Improved endoscopic imaging tools have a uniquely suited role with the advent of endoscopic ablative therapies. GI mucosal diseases can be treated using endoscopic mucosal resection (EMR) [50,51] or endoscopic ablative therapies, such as argon plasma coagulation (APC) [52], photodynamic therapy (PDT) [42], radiofrequency ablation (RFA) [53], and cryospray ablation (CSA) [54]. RFA and CSA are recently developed methods that utilize thermal gradients to treat dysplastic or cancerous GI lesions.

RFA uses heat generated by high frequency alternating current to treat the diseased area and has been demonstrated as an effective treatment for dysplastic BE. Of 142 patients with high grade dysplasia (HGD) undergoing circumferential RFA, Ganz, *et al.* reported complete response in eradicating HGD in 90.2% and complete response eradicating all dysplasias in 80.4% [55]. Sharma *et al.* reported 70% and 98% complete response in BE patients at 12 months and 30 months follow-up respectively after RFA treatment [56,57]. Pouw *et al.* reported 98% complete histological eradication of all dysplasia and intestinal metaplasia in 44 BE patients treated with RFA [58]. One randomized trial using RFA in BE patients with dysplasia has also been published by Shaheen *et al.* and 77.4% complete eradication of intestinal metaplasia over 12 months was reported [53].

CSA, on the other hand, uses dispersed liquid nitrogen to rapidly freeze and destroy the diseased tissue. CSA has also been shown to be effective in eradicating HGD in the esophagus. Johnston *et al.* reported 78% complete histological eradication of BE in 11 patients using CSA [54]. Greenwald *et al.* reported 94%, 88%, and 53% complete response of the HGD, all dysplasia, and intestinal metaplasia, respectively, in a cohort of 77 patients with BE treated by CSA [59]. Shaheen *et al.* reported 97%, 87%, and 57% complete eradication of HGD, all dysplasia, and intestinal metaplasia in 98 BE patients treated with CSA [60].

Several studies indicate that both RFA and CSA allow broad and superficial treatment fields for GI diseases [53,54,61,62], but some other studies show that the recurrences of the diseases were observed months or years after successful eradication. Vaccaro *et al.* reported a 25.9% cumulative incident of newly detected intestinal metaplasia in one year [63]. Fleischer *et al.* reported 8% BE recurrence in 50 patients who had complete eradiation of BE during the five-year follow-up in a prospective multicenter trial [64]. There are several possible causes that the recurrence of the GI diseases can occur after the complete eradication. One postulate is that, after the ablation therapies, the ablated sites recover with neosquamous epithelium covering on the top. A significant portion (20%–30%) of patients show subsquamous intestinal metaplasia (sometimes referred to as “buried glands”, or SSIM) under the neosquamous epithelium after the therapeutic treatment based on random biopsies [65,66], which could be associated with a future risk of recurrence of BE or progression to adenocarcinoma under the neosquamous epithelium [52,67]. Currently, random four-quadrant biopsy is the clinical standard for evaluating dysplasia and adenocarcinoma in BE and colorectal cancer patients [68,69]. Due to the limitation sampling area (about 1–2 mm^2^) and sampling depth of biopsy forceps, SSIM was only found in 25% of patients before and in 5% of patients after successful RFA treatment [53,60,62,64]. A technology such as 3D-OCT that can be used for guiding excisional biopsy and providing subsurface tissue imaging would significantly reduce sampling errors, improving diagnostic sensitivity, treatment follow-up, and outcome.

## 3. Development of Endoscopic OCT

OCT technology has been successfully applied to numerous biomedical fields including ophthalmology [70], cardiology [71], gastroenterology [72], urology [73], and gynecology [74]. Applications for an ultrahigh speed 3D-OCT imaging are particularly promising in gastroenterology. OCT can be readily integrated with a wide range of imaging devices such as fiber optic catheters and endoscopes to enable imaging inside the body [6]. The first demonstration of *in vivo* endoscopic OCT was performed by Tearney *et al.* [6]. This study demonstrated high speed OCT imaging of the gastrointestinal and pulmonary tracts in the rabbit using a 1mm diameter fiber optic catheter. The earlier development of endoscopic OCT technology can be found in the detailed review by Yaqoob *et al.* [75].

### 3.1. Earlier Clinical Studies Using Endoscopic OCT

Endoscopic OCT imaging of the human gastrointestinal (GI) tract has been investigated by several groups and studies have been performed in the esophagus and stomach [72,76,77,78,79,80,81,82,83], small and large intestine [77,79,84,85,86,87,88], and bile duct [89,90]. Barrett’s esophagus was clearly differentiated from non-neoplastic tissues. OCT has been demonstrated to detect specialized intestinal metaplasia in BE patients [91,92] and transmural inflammation in IBD patients [85]. OCT has also been investigated for differentiating hyperplastic from adenomatous polyps in the colon [86]. Recent endoscopic OCT studies have shown promise for detection of HGD in BE. Evans, *et al.* reported a sensitivity of 83% and a specificity of 75% for detecting HGD and intramucosal carcinoma with blinded scoring of OCT images from 55 patients [93]. Isenberg *et al.* reported a sensitivity of 68% and a specificity of 82%, with an accuracy of 78% for the detection of dysplasia from 33 patients with BE [94]. Using computer-aided tissue classification techniques applied to OCT images, Qi *et al.* reported a sensitivity of 82% and a specificity of 74% for identifying dysplasia in 13 patients [95].

### 3.2. Important Advancement Using High Speed Fourier Domain OCT

With the development of Fourier domain OCT technologies, endoscopic OCT imaging has recently been demonstrated with much higher imaging speed and better resolution. Ultrahigh resolution spectral/Fourier domain endoscopic OCT with 2.4 µm axial resolution using a Ti:Sapphire laser with 20 kHz axial scan rates was demonstrated in the mouse colon [96]. Using swept source/Fourier domain technologies, *in vivo* 3D-OCT volumetric imaging of the porcine esophagus and artery has been demonstrated at 10 kHz and 54 kHz axial scan rates, respectively [97,98]. Using FDML technologies, Prof. Fujimoto’ s group at MIT achieved record axial scan rates of 100–500 kHz and demonstrated *in vivo* endoscopic 3D-OCT imaging in the rabbit and human GI tracts with axial resolution of 7–20 µm [29,99]. Suter *et al.* demonstrated comprehensive volumetric endomicroscopy of the human distal esophagus using 3D-OCT and a balloon-centering imaging catheter, providing a wealth of detailed information about different structural features of the normal esophagus, esophageal specialized intestinal metaplasia, high grade dysplasia, and intramucosal carcinoma [100]. These endoscopic studies demonstrate that OCT imaging can be readily integrated with endoscopic procedures and potentially provides valuable diagnostic information. There are still very few clinical studies using endoscopic OCT in the GI tract that provide significant solutions to certain clinical problems, and only recently were such instruments commercially available, so currently OCT remains largely an investigational tool not accepted as a standard imaging modality for most GI clinics.

### 3.3. Comparison between Different Endoscopic Imaging Technologies

Several advanced imaging technologies have been developed to increase the yield of the endoscopy surveillance. Chromoendoscopy with topically applied dyes such as methylene blue or indigo carmine over the regions of interest facilitates the detection of nonpolypoid lesions based on the enhancement of surface morphology [101,102,103,104], but the main limitations of chromoendoscopy are the requirements of high quality bowel preparations for standard colon screening, large volume of dye, time required for dye uptake, and need for copious flushing [105,106]. Virtual chromoendoscopy such as narrow-band imaging (NBI), where light of specific blue or green wavelengths is used, can enhance the detail of certain aspects of the tissue surface such as blood vessels without any application of dye [107,108]. Although chromoendoscopy and NBI are able to enhance the tissue contrast and distinguish lesions from normal tissue, the optical resolution of these methods with standard endoscope is not sufficient to distinguish dysplasia from non-dysplastic tissues. Recent studies show that these ancillary imaging modalities do not enhance detection sensitivity or specificity over standard white light endoscopy in general clinical practice [109,110]. In conjunction with high-magnification endoscope, which has not yet been widely available in the United States, it is possible to use chromoendoscopy or NBI to visualize different pit patterns of the lesion and detect dysplasia in the colon based on the Kudo classification [111] and in the esophagus based on the Paris classification [112,113]. Confocal laser endomicroscopy (CLE) is another emerging technology that allows microscopic examination of the GI tract and has been demonstrated having high sensitivity and specificity in differentiating neoplasia in both esophagus and colon [114,115,116,117,118,119]. CLE requires the use of exogenous contrast agents such as intravenous fluorescein and topical acriflavine (not approved for human use in the United States), so the time for imaging is limited due to excretion of fluorescein [120,121]. The high magnification of CLE also limits its field of view (FOV) and imaging depth, making it impractical to survey large areas for possible malignancy or to detect important features in the deeper tissue regions. Table 1 shows a comparison of features between these endoscopic imaging technologies. Among these imaging modalities, endoscopic OCT can uniquely provide volumetric structural information in the GI tract and is well-suited for early GI cancer detection including screening and surveillance. Recently, several “red flag” techniques, such as the application of fluorescence-labeled peptides that can specifically bind the dysplastic regions [122,123,124,125] and angle resolved low coherence interferometry (a/LCI) that uses depth-resolved nuclear morphology measurements to detect dysplasia [126,127,128,129], also showed promising results in identifying dysplastic tissue over a large field in the GI tract. These techniques, used solely or in conjunction with OCT, can potentially be very powerful once they are validated with large clinical studies in the future.

**Table 1:** Comparison of endoscopic imaging modalities.

Imaging Modality	Depth	Axial Resolution	Pros	Cons
Ultrasound	1–10 cm	50 µm	Broad field Standard for staging tumors despite improvements in CT and MR	Location of neoplasia Low resolution
Chromoendoscopy	N/A	N/A	Enhanced endoscopic imaging contrast	Superficial imaging Large volume dye required
NBI	N/A	N/A	Enhanced endoscopic imaging contrast No contrast agent required	Superficial imaging
CLE	<250 µm	1–5 µm	Cellular resolution Point and view	Limited imaging depth Limited field of view IV contrast required
LCI	200–300 µm	N/A	Assessment of nuclear size Red flag technology No contrast agent required	Limited detection depth
OCT	3 mm	5–30 µm	No contrast agent required 3D visualization of tissue structure	Can’t image fluorescence 3D presently not real-time

### 3.4. Imaging Catheter Designs Using Different Scanning Mechanism

Generally speaking, there are two main categories of scanning methods used in imaging catheters: proximal actuation and distal actuation. The imaging catheter with proximal actuation usually contains actuators in the patient interface unit (PIU) outside of the human body and far away from the tissue. The actuation is usually transferred to the distal end of the imaging catheter via a torque coil or a guide wire. Fast axis scanning can be done using high speed rotary or push-pull methods. To acquire volumetric OCT data set, slow axis scanning is also required. High speed rotary plus slower pulling back of the probe is the most common method to acquire 3D OCT data sets [97,98,100,130,131,132,133].

**Figure 4:**
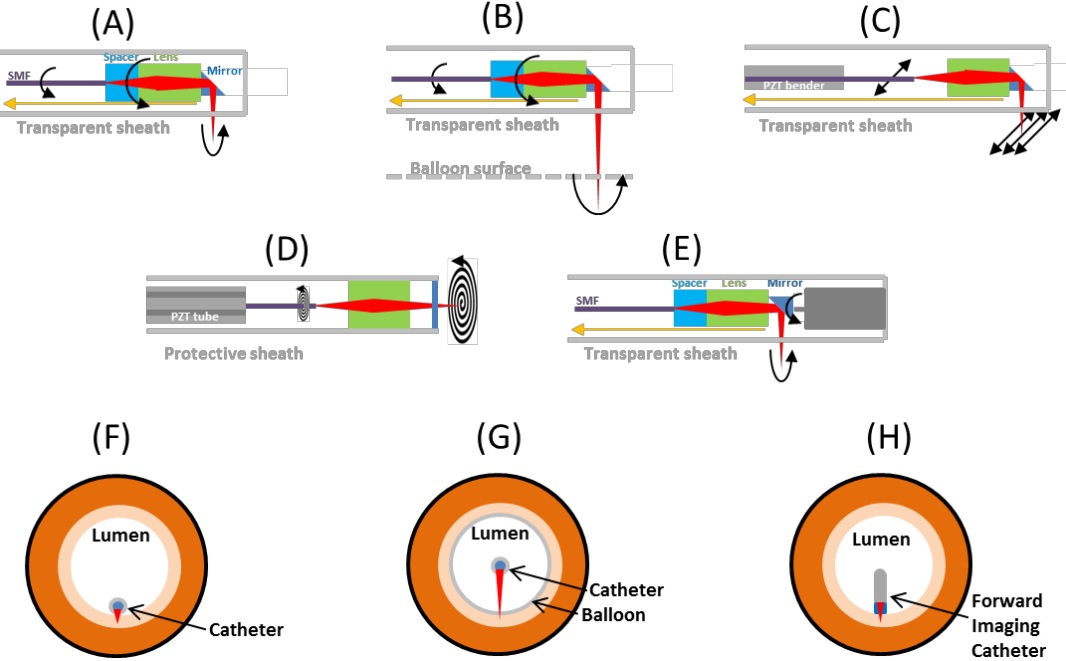
Schematics of different scanning methods. (**A**) Standard rotary scan with pull back. (**B**) Rotary scan with balloon and pull back. (**C**) Raster scan perpendicular to axis with rotation and pull back. (**D**) Spiral scan. (**E**) Micromotor rotary scan with pullback. (**F**) Front cross-sectional view of the imaging catheter in the lumen for scanning method (**A**), (**C**) and (**E**). (**G**) Front cross-sectional view of the imaging catheter in the lumen for scanning method (**B**). (**H**) Front cross-sectional view of the imaging catheter in the lumen for scanning method (**E**) and (**D**). Black arrow: fast scan. Yellow arrow: slow scan.

On the other hand, distal actuation uses an actuator placed at the distal end of the imaging catheter. Hence, the actuator must be made as small as possible to go through the working channel of an endoscope or used alongside the endoscope without increasing the overall size too much. There are several miniaturized actuators used in distal actuation probes such as piezoelectric transducer (PZT) based devices [134,135,136] and microelectromechanical system (MEMS) based devices [137]. Miniaturized actuators can achieve either one-dimensional (1D) or two-dimensional (2D) scanning. The actuators are usually controlled electronically and are very close to the imaging surface, so electrical isolation is important in these imaging catheters. Figure 4 shows schematics of these different scanning methods, including: (1) proximally rotary (short working distance) side imaging, (2) proximally rotary balloon (long working distance) imaging, (3) raster scan side imaging, (4) raster forward imaging, and (5) spiral forward imaging.

#### 3.4.1. Proximal Rotary Side Imaging

This is the scanning method used in most of the studies conducted with endoscopic OCT imaging, which was first developed by our group at MIT in 1996 [130], as shown in Figure 4A. Rotation is produced by a high speed motor and transferred to the distal end of the imaging probe via a torque coil. Slow scan is performed by slowly pulling back the whole probe to achieve 3D data acquisition. This design requires a fiber optic rotary joint so the imaging catheter can continuously rotate in one direction. The rotary joint typically has maximum rotary speed of 2000–10,000 revolutions per minutes (rpm), corresponding to 33–166 revolutions per second, so the frame rate limit of this method is around 150 frames per second (fps). Since the light is always on axis in the optics, the effect of spherical aberration effect (off axis light is focused at different focal plane) is small. Therefore, this method is easier for optical design and the beam profile is not distorted due to scanning. Moreover, the distal end can be made very small and the rigid length can be short with the simple optic design. Typically the outer diameter (OD) of entire probe including sheath can be thinner than 2 mm and the rigid length can be less than 2 cm, allowing passage through the accessory channel of most standard endoscopes. This proximal rotary method requires the rotation of the whole imaging catheter during the imaging, so the scanning may be hindered by the bending of the imaging catheter, resulting in non-uniform rotation of the distal imaging optics and limiting the image quality even if the transverse optical resolution is high. The scanning speed can be limited because the torque coil can vibrate when operated at rotary speed higher than a few thousand revolutions per minute (rpm). The field of view (FOV) is also limited by the size of the catheters due to the necessity of keeping the catheter close to the tissue surface. For example, for an imaging catheter of 2.5 mm OD, the scanning range provided by the catheter is about 8 mm, which is roughly 1/10 of the average circumference of adults’ esophagus. Hence, this type of catheter is suitable for applications that focus on relatively small regions of interest in the GI tract.

#### 3.4.2. Proximal Rotary Balloon Imaging

In order to reduce the environmental instability of the high speed rotary probe, balloon probes were adopted to stabilize the probe in the human body, especially for studies in the GI tract [100], as shown in Figure 4B. With the balloon design, it is easier to image the entire circumference of the esophageal lumen around the imaging catheter and co-register the imaging locations at different time points. The balloon increases the working distance (~1 cm) from the optics to the lumen and increases the size of the probe, but keeps the spot size on the imaging plane small enough to maintain reasonable transverse resolution. With the longer working distance, the aberration of the focal spot caused by any curved interface in the catheter is more severe compared to the non-balloon design, so additional corrective optical designs are required. Commercial endoscopic OCT instruments such as the NvisionVLE imaging system (NinePoint Medical) have recently been introduced which use balloon imaging catheters.

For single balloon designs, the balloon exerts pressure to the surrounding tissue and may deform the tissue structure such as flattening potential cystic structures and compressing the thickness of layers (epithelium, lamina propria, muscularis mucosa, *etc*.) [100]. A double balloon design, which places the imaging probe between the two balloons, can solve the deformation issue, but the imaging location in the human body will be limited [133]. For example, a double balloon catheter may not be appropriate at the gastroesophageal junction (GEJ) because the distal balloon would be in the stomach and cannot stabilize the imaging catheter. One general disadvantage of the balloon design is that the probe might not always be at the central position of the balloon, so some part of the tissue can be out of the imaging range, even if the probe is well stabilized in the lumen.

#### 3.4.3. Raster Scan Side Imaging

Proximally actuated imaging catheters generally have the problem that the scanning is not uniform, particularly with increased resistance on the torque coil from external compression or bending during endoscope articulation. The scanning speed is also limited because the torque cable tends to vibrate when operated at rotary speeds higher than a few thousand revolutions per minute. This limits the ability to obtain detailed structural information and smaller features due to the heartbeat or breathing induced motion artifact and lack of registration between sequential images in the pullback. Hence, distally actuated scanning probes are preferable for visualizing small features of the tissue. One method used for distal actuation involves sweeping the fiber tip at the distal end of the imaging catheter either by a PZT or electromagnet actuators to achieve raster scanning [29]. The beam from the fiber tip is then focused by a lens and can be deflected by a prism or mirror. The scanning speed of PZT based benders can easily achieve over 1 kHz if the total length of the actuator is short, while the scanning range is limited because the scanning range is proportional to the square of the length of actuator [29].

An important issue for the scan is the imaging plane distortion caused by the aberration of the lens and sheath when the light is scanned away from the optical axis. A compound lens or GRIN lens is preferable because it can correct the aberration caused by a single lens [138]. Also, the distortion by the curvature of sheath can affect the beam quality while scanning to off axis position. Raster scanning perpendicular to the imaging catheter (Figure 4C) has high aberration because the beam is incident with a large angle on the curved surface of the probe sheath.

#### 3.4.4. Spiral Scan Forward Imaging

Forward imaging using quadruple PZT tubes was demonstrated several years ago [134] as a method for small diameter endoscopic imaging and is also a feasible way to acquire endoscopic OCT images [135]. As Figure 4F shows, the fiber goes through the PZT tube and can be actuated two-dimensionally. By applying amplitude-varying sinusoidal waves with 90° phase difference to the two pairs of the electrodes, the fiber tip can be actuated in a spiral pattern and obtain 3D OCT data. Since both axes are actuated distally, the scan can be controlled precisely so this scanning mechanism is particularly suitable for the high magnification, small field imaging, such as optical coherence microscopy (OCM) and multi-photon microscopy (MPM). Recently, a new method which drives two scanning directions with slightly different frequencies has been described [139]. The resulting scanning pattern is a Lissajou pattern with the frame rate of the difference of the two driving frequencies. These methods eliminate the need for an additional slow scan, so the probe design can be less complex and its size can be smaller than the raster scanning probe.

However, these scanning methods require very precise mapping of the pixels during the scan. The scanning density of the spiral and Lissajou patterns are also non-uniform. Another issue for the scanning method is that the fiber tip is driven with frequencies close to its resonance. Any environmental perturbation or impact that contains the frequency components close to the resonance frequency of the fiber while imaging could couple into the motion of the fiber tip and disrupt the scanning pattern and pixel mapping. This is a common problem for raster, spiral, and Lissajou methods because they both vibrate the fiber with frequency close to resonance.

Table 2 summarizes and compares these scanning methods. Depending on the application, a specific scanning mechanism with suitable parameters of scanning range, scanning speed, and number of pixels in a frame can be used. In addition to the above mentioned scanning mechanisms, imaging using distal rotary scanning can be achieved with advances in micromotor technology, which can provide large scanning areas while maintaining high speed and uniform rotation. Micromotor catheters for ultrahigh resolution *in vivo* OCT imaging were demonstrated in 2004 for imaging at 2 fps with 1000 axial scans per frame [140,141]. Micromotor catheters have been used in upper airway [142] and intravascular imaging [143]. Recently, several research groups have demonstrated ultrahigh speed endoscopic OCT imaging with imaging speed ranging from 52 to 3200 fps in *ex vivo* specimens [144,145], and 400 fps in the rabbit *in vivo* [146].

**Table 2:** Comparison between different scanning mechanisms.

Scanning Mechanisms	Advantage	Disadvantage
Proximally rotary	Simple optical design Small No aberration Moderate scanning range (1–4 cm^2^) Water can be used to reduce reflections/aberration	Rotary non-uniformity Pull back non-uniformity Unstable slow scan
Rotary with single balloon	Simple optical design Small More stable for slow scan Large scanning range (>30 cm^2^) Cover the whole lumen circumference Water can be used to reduce reflections/aberration	Rotary non-uniformity Deformation of tissue Probe off-centered Aberration from sheath
Rotary with double balloon	Simple optical design Small More stable for slow scan No tissue deformation Large scanning range (>30 cm^2^) Cover the whole lumen circumference Water can be used to reduce reflections/aberration	Requires balloon and inflation Rotary non-uniformity Probe off-centered Aberration from sheath Imaging location limited
Raster scan side (perpendicular)	Higher scanning speed Moderate scanning range No fast scan non-uniformity	Image area is long strip Larger probe Sinusoidal wave correction Sensitive to perturbation Aberration from scanning at high angle to sheath Water cannot be used
Spiral/Lissajou forward scan	Small Simple design No fast scan non-uniformity Simultaneous 2D scan	Limited imaging range Scanning pattern correction Sensitive to perturbation Slower scanning speed
Micromotor	Highest scanning speed No fast scan non-uniformity Large scanning range Could incorporate with other design	Proximal pullback required Field of view blocked by electric cables

For all the catheter designs, a major practical issue that needs to be considered is the overall catheter dimensions, including the outer diameter and the rigid length. These two dimensions determine whether an imaging catheter can go through the working channel of the endoscope. The typical diameter of the endoscope working channel is between 2.8 and 3.7 mm. Although there are some endoscopes with larger working channels (>5 mm diameter), the ~30 degree turn in the insertion port of the working channel still limits the diameter of the imaging catheter that can go through the channel. Hence, if the imaging catheter needs to go through the working channel of conventional endoscope, the preferable catheter diameter should be less than 2.5 mm and rigid length less than 25 mm. This limits the components, including optics and actuators, which can be used to develop the imaging catheters. For most of the proximal actuated imaging catheter, these dimension requirements are easy to meet because there is no scanning part in the distal end. The other insertion method is the “overtube” configuration. Rather than going through the working channel of the endoscope, the imaging catheter is attached outside of the endoscope tip. This allows the imaging catheter to be made in a larger size and with more component options, which helps to achieve better imaging resolution and more stable scanning. However, the overall diameter of the imaging catheter should not be larger than 5 mm. Otherwise, it is very difficult to insert the endoscope into the human body with the catheter attached. To date, there is no endoscopic OCT study that uses the overtube configuration.

## 4. Recent Achievements of Endoscopic OCT in Clinical Gastroenterology

Endoscopic 3D-OCT imaging has been performed extensively on patients with BE or IBD, and several case reports and clinical studies have been published recently. This section will provide a brief overview of representative studies conducted by different groups using state-of-the-art endoscopic OCT system in conjunction with the white light endoscopy, which indicate the unique utility of endoscopic OCT in clinical gastroenterology.

### 4.1. Laser Marking and OCT Guided Biopsy

The gold standard method to detect dysplasia and intramucosal carcinoma in Barrett’s patients is to perform random, 4-quadrant endoscopic biopsy of the involved esophageal segment. Due to the limited overall coverage of random biopsy procedure over the involved tissue, this procedure can lead to a high sampling error for detecting malignancy [147]. Endoscopic OCT can provide cross-sectional images of the esophagus with a resolution of about 5–30 µm and can accurately detect various esophageal pathologies [92,93,94]. Previously, OCT was able to image only small areas of the esophagus at a time owing to limitations of image acquisition speed and catheter scanning mechanism. By using a balloon-based catheter that extends the working distance of the OCT imaging and a spiral scan, 3D-OCT covers a much larger area on a single sweep and enables comprehensive microscopy in the distal esophagus within minutes [98,100]. The ability to image the entire esophagus at microscopic resolution makes OCT particularly suited for guiding biopsies. Suter *et al.* developed an OCT imaging system that allows superficial and readily visible laser markings on the mucosa corresponding to regions of interest on the images to guide biopsies and ablative therapies immediately following imaging [83].

**Figure 5:**
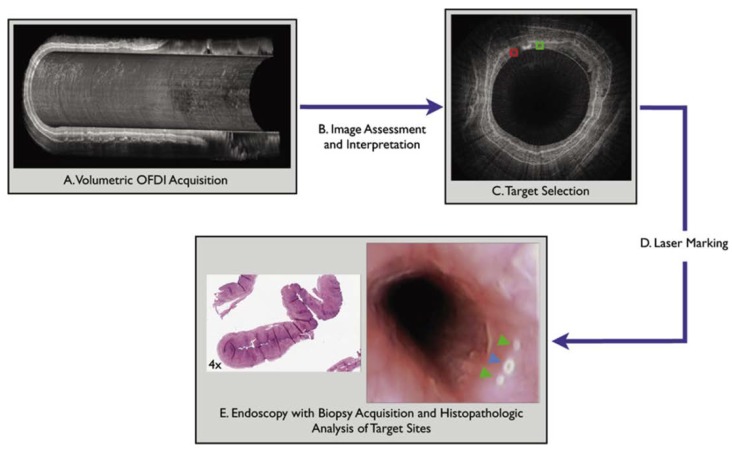
Guided biopsy with the use of laser marking and endoscopic OCT imaging. (**A**) volumetric OCT imaging of the region of interest in the esophagus; (**B**) assessment and interpretation of the volumetric data set; (**C**) selection of point of interest; (**D**) laser marking at corresponding sites on the luminal wall; and (**E**) endoscopic biopsy at the marked sites for histopathologic analysis. Originally published in [83].

Figure 5 outlines the steps for directed biopsies using the laser-marking OCT, as validated in a recent study using swine. In the study, the balloon catheter was placed in the esophagus alongside the endoscope and after positioning, the endoscope was removed and the balloon was inflated. The balloon catheter dilated the esophagus and stabilized the imaging probe with respect to the esophageal wall. After the laser marking, the endoscope was reintroduced to evaluate the visibility of the laser-marked sites. The feasibility of the OCT-guided laser marking was evaluated by determining the optimal laser parameters, testing the accuracy of the laser marking process, evaluating the endoscopic visibility of the laser marks, and assessing the amount of mucosal damage produced by the laser.

This study showed that all of the laser-induced marks were visible by endoscopy and 97% of the target locations were successfully marked. Laser marking in conjunction with OCT imaging was demonstrated to be effective for guiding endoscopic biopsy, could potentially enhance the diagnostic yield of current endoscopic surveillance protocols, and may provide assistance during other interventional treatments such as identifying margins for EMR, as well as with co-registration of the endoscopic images and histology. Combined with the laser marking technology, the real-time imaging capability of endoscopic OCT is extended to actively mark down the points of interest accurately and can be used to guide biopsy *in vivo*.

### 4.2. Cancer Classification

**Figure 6:**
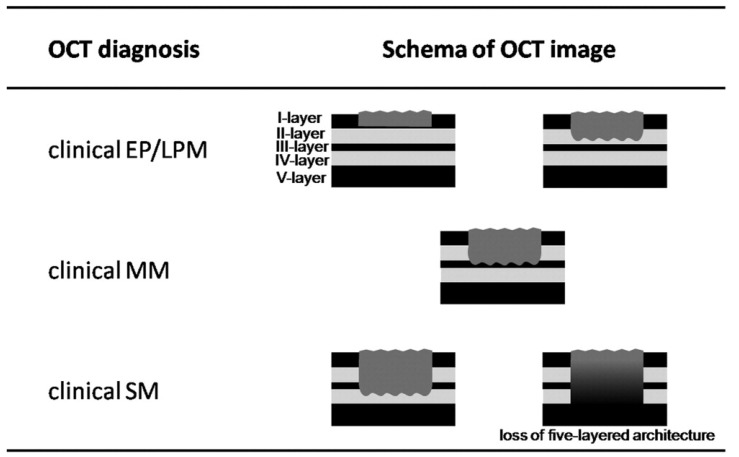
OCT imaging criteria for the staging of SESCC. The dark gray area indicates tumor. Originally published in [151].

The use of endoscopic resection (ER) is limited to carcinoma invading the lamina propria layer according to the current esophageal cancer management guidelines [148], and surgery or chemoradiotherapy is performed for tumor invasion to the muscularis mucosa or deeper layer. Endoscopic ultrasound (EUS) has been suggested as a useful method for the evaluation and staging of tumor infiltration in superficial esophageal squamous cell carcinomas (SESCCs) [149], but the accuracy of EUS is not sufficient due to the poor resolution [84]. Endoscopic OCT is analogous to B-mode ultrasound, but can produce higher-resolution cross-sectional images of tissue in real time because it uses infrared light that has much shorter wavelength compared to ultrasound. This results in approximately 10-fold better axial resolution images that can identify tissue microstructures, such as layered structure in the mucosa and glandular structures [94], and may allow more accurate diagnostic assessment of tumor invasion in SESCCs. In normal pig esophagus, the layered structure observed in OCT images was demonstrated to correspond to histological structures [150], namely squamous epithelium (EP), lamina propria (LP), muscularis mucosa (MM), submucosa (SM), and muscularis propria (MP). Hatta *et al.* performed a clinical study to establish image-based criteria for staging tumor infiltration in SESCCs and to evaluate the accuracy of these criteria [151]. In a single center, prospective two-phase study, 66 patients with SESCC were enrolled to participate. Using a prototype OCT system developed by Lightlab Imaging, Inc. (Westford, MA, USA) and HOYA (Tokyo, Japan), 35 OCT images from 16 patients were evaluated in the first phase of the study to establish the OCT image criteria of three categories (Figure 6) based on SESCC involvement of the layers. In the second phase of the study, 109 images from 46 subsequent consecutive patients were used for prospective evaluation of the criteria. The OCT images were reviewed and staging was assessed by one experienced gastroenterologist blinded to endoscopic evaluation and other clinical information. The accuracy of the OCT criteria was determined by comparing the OCT-based staging results with the histological evaluation of the resected specimens. The overall accuracy to identify the depth of the cancer invasion based on OCT images was 92.7% (101/109), and the accuracy of cancers that reach EP/LP, MM, and SM layers was 94.7% (74/78), 85.0% (17/20), and 90.9% (10/11), respectively. The OCT imaging criteria developed for staging tumor invasion was demonstrated to accurately stage SESCCs and can be useful in determining endoscopic resectability. However, this study also pointed out the limitations of OCT, including the limited image penetration, inability to discern cancer cell invasion and inflammatory cell infiltration, and inability to discriminate the cancer within EP layer alone from normal esophageal tissue. These limitations may be overcome by further development of OCT technology to provide deeper image penetration, higher resolution as with the ultrahigh-resolution OCT [152], and functional imaging as with Doppler OCT [153].

### 4.3. Investigation of Subsquamous Intestinal Metaplasia

**Figure 7:**
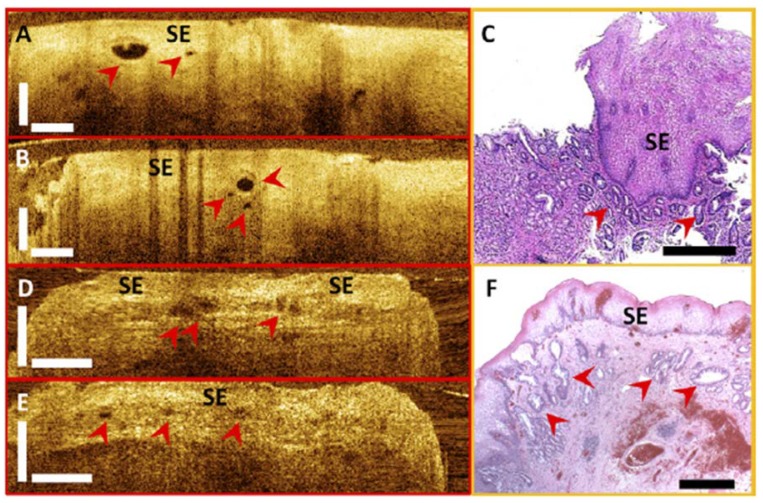
Cross-sectional optical coherence tomography images and corresponding histology showing buried glands (red arrowheads) from a patient *in vivo* (**A**–**C**) and an *ex vivo* EMR specimen (**D**–**F**). (**C**,**F**) Histological micrographs confirmed the presence of buried glands with intestinal metaplasia. SE: squamous epithelium. Scale bars, 500 µm. Originally published in [155].

Subsquamous intestinal metaplasia (SSIM), also known as buried glands, is usually underappreciated with current surveillance protocols using standard white light endoscopy [154]. Endoscopic 3D-OCT imaging uniquely enables depth-resolved imaging of a broad area with near-microscope resolution and can be used for identifying and characterizing SSIM before and after ablative therapies [82]. Figure 7 shows examples of cross-sectional OCT images and corresponding histology. Figure 7A and Figure 7B were acquired *in vivo* at the position of the GEJ. SSIM was identified with 3D-OCT at the GEJ of a patient during the imaging procedure. The corresponding histological micrograph shown in Figure 7C confirmed the presence of SSIM with intestinal metaplasia underneath the neosquamous epithelium. Figure 7D and Figure 7E were obtained from an *ex vivo* EMR specimen to enable more accurate correspondence of OCT images with histology. From cross-sectional OCT images, squamous epithelium was characterized by a low scattering, homogeneous layer at the mucosal surface. SSIM was identified as sparsely distributed hyposcattering structures underneath the squamous epithelium with various sizes and shapes. The histological micrograph of the EMR specimen (Figure 7F) also confirmed the presence of buried glands with intestinal metaplasia.

In the study of 27 patients with short-segment BE, 3D-OCT was demonstrated to provide a 30–60 times larger field of view compared with jumbo and standard forceps biopsy and sufficient imaging depth to the lamina propria/muscularis mucosa to facilitate the detection of SSIM before and after the treatment [155]. A high prevalence of SSIM was found in 72% of patients who had residual BE and in 63% of patients who achieved complete response to the ablation treatment. The high prevalence is consistent with biopsy results from previous studies when accounting for difference in sampling area between OCT imaging and excisional biopsy [156]. The number of SSIM per patient decreased significantly after the patients achieved complete response (*p* = 0.02). However, the size and distribution of the SSIM did not change significantly in patients with short-segment BE whether the patients achieved complete response or not. This study suggested that OCT may be used to stratify the patient risk and could help direct areas for re-ablation.

### 4.4. RFA Treatment Response Evaluation

Repeated RFA treatments are generally required to achieve complete eradication of intestinal metaplasia. Complete eradication of intestinal metaplasia (CE-IM) was achieved over three sessions on average for patients with BE [53,64,157]. The long overall treatment period plus the cost to achieve complete eradication mounts with each esophagogastroduodenoscopy (EGD) and RFA procedure, which has tempered the enthusiasm to treat patients with non-dysplastic BE (NDBE) using RFA [64]. There are various causes that may contribute to incompleteness of the ablation. Since the dosage of the RFA treatment has been set to achieve best efficacy with minimum injury depth [158,159], the energy delivery of a standard RFA application might not reach deep enough when the BE epithelium is thick. The variation of the RFA electrode contact with the esophagus and coagulated debris building up on the electrode surface during repeated ablation in one patient also can degrade the effectiveness of the radiofrequency energy delivery [159]. Therefore, tissue structural features, including SSIM, the BE epithelium thickness, and residual glands that receive insufficient ablation or no ablation, can potentially be used to predict or evaluate the ablation treatment response. It is difficult to evaluate the presence of residual glands or unburned BE by using white light endoscopy or narrow-band imaging because of the limited visibility immediately after the RFA treatment, when the endoscopic imaging field is covered with blood and tissue debris. Traditional evaluation of treatment response is usually performed after 6–8 weeks allowing the recovery of the ablated regions, so the entire treatment process can easily span half a year or longer if multiple RFA sessions are required.

**Figure 8:**
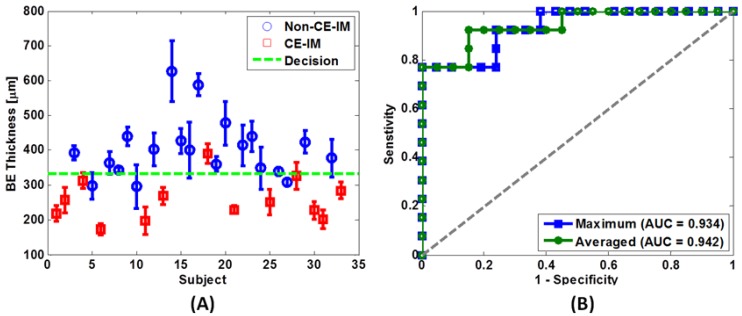
(**A**) Scatter plot of the average BE epithelium thickness measured by OCT from different patient groups. Blue circles: patients who did not achieve CE-IM at the follow-up sessions; Red crosses: patients who achieved CE-IM at the follow-up sessions; Green dotted line: discrimination threshold at 333 µm as determined from the average BE thickness ROC curve in (**B**). (**B**) ROC curves of treatment response prediction using average (green) and maximum (blue) BE thickness. The area-under-the-curve (AUC) values were 0.942 (*p* < 0.001) and 0.934 (*p* < 0.001) using the average and maximum BE thickness, respectively. Originally published in [160].

In the study of 33 patients with short-segment BE, 3D-OCT identified structural markers, including the thickness of the BE epithelium prior to RFA and the presence of residual glands immediately after RFA, which might be used to predict RFA treatment response at follow-up with high accuracy [160]. It was found that BE thick varied widely, from 200 to 700 µm in this cohort of patients, and OCT measurement of BE thickness of >330 µm predicted incomplete treatment response at follow-up visit 6–8 weeks after the RFA treatment with a sensitivity and specificity of 92% and 85%, respectively (Figure 8A,B). This raises the possibility that energy delivery of a standard RFA application may not be reaching deep enough when the BE epithelium is thick. In this study, a correlation was found between the BE epithelium thickness and the presence of residual glands immediately after RFA, suggesting not all RFA applications were effective. Insufficient energy delivery at the treatment sites or leaving BE unburned may be factors that lead to endoscopically visible residual BE at follow-up.

The efficacy of ablation therapies is assessed by follow-up endoscopy 6–8 weeks after RFA, and patients undergo repeated ablation if residual BE is observed. These results suggest that patient complete response rate may be stratified based on the BE thickness measured before RFA. Using this structural marker, it may be possible to adjust RFA treatment for each patient to optimize response. For patients with thicker BE epithelium, a more rigorous ablation may be required. This may involve a removing debris from the ablation catheter and treatment areas between the two sets of ablations, and if indicated, changed dosage for the RFA treatment. The ability of OCT to differentiate residual glands from normal tissue structures and debris caused by the ablation may also provide immediate feedback on the RFA treatment for the endoscopist. This may enable “smart ablation” mechanism that real-time evaluates the ablation depth and identifies regions requiring further treatment. The RFA treatment might be guided to further improve the efficacy of each ablation procedure. As a result, the number of treatment sessions might be reduced to reduce overall treatment time, patient anxiety and health care cost.

### 4.5. Investigation of Cervical Inlet Patch

Cervical inlet patch (CIP) is characterized by the presence of heterotopic columnar gastric mucosa in the upper esophagus, most commonly located just below the upper esophageal sphincter (UES). The incident of CIP has been reported from as low as 1% to as much as 10% of endoscopic cases in different adult studies [161,162], but is under-appreciated given its location generally just below the upper esophageal sphincter, which remains an area of poor detection during routine upper endoscopy. It is unclear whether CIP is congenital or acquired. One postulate is that CIP originates from incomplete embryonic replacement of the stratified epithelium, which normally starts at the fourth month of gestation. Immunohistochemical studies suggest an embryologic origin for CIP on account of differences in endocrine markers such as serotonin, glucagon, pancreatic polypeptide, somatostatin and neurotensin in histologic specimens of CIP and BE [163]. A second postulate is that CIP, especially as noted in adults, is an acquired metaplastic change occurring in the squamous mucosa of the esophagus and is associated with predisposing factors for gastroesophageal reflux disease (GERD), such as sliding hiatal hernia [164]. Its incidence is up to four-fold higher in patients having BE [165] and CIP was found in almost one third of patients having dysplastic BE or adenocarcinoma [166]. Thus, long-standing acid reflux is thought to lead to columnar metaplasia in the upper esophagus, similar to BE. Several reports suggest that CIP may progress to adenocarcinoma [167,168,169,170].

**Figure 9:**
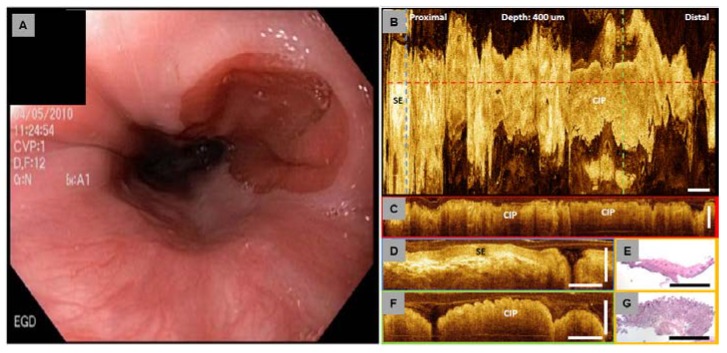
Cervical inlet patch (CIP). (**A**) Endoscopic view. (**B**) *En face* projection OCT image at 400 µm underneath the tissue surface. Regions with CIP and surrounding squamous epithelium (SE) can be identified. (**C**) Cross-sectional OCT image along the probe pullback direction, corresponding to the red dashed line marked in (**B**). (**D**,**F**) Cross-sectional OCT images of squamous epithelium and CIP, respectively. The OCT images correspond to the blue and green dashed lines marked in (**B**), respectively. (**E**,**G**) Representative histology of normal squamous epithelium and CIP. Scale bars: 1mm. Originally published in [171].

Figure 9 shows an endoscopic view of and volumetric 3D-OCT data set of CIP obtained from a 30-year-old patient [171,172]. During retraction of the endoscope, a pink circular lesion was observed under white light endoscopy in the upper esophagus (Figure 9A). The *en face* projection image (Figure 9B) at 400 µm depth underneath the tissue surface showed columnar epithelium consistent with the CIP and surrounding squamous epithelium. Cross-sectional OCT images (Figure 9C, Figure 9D, and Figure 9F) clearly demonstrated columnar and squamous epithelium in the CIP region and the surrounding esophagus, respectively. Biopsy specimens taken from the imaged lesion confirmed the finding of CIP. The OCT features matched representative hematoxylin and eosin (H&E) histology (Figure 9E and Figure 9G). These results demonstrated the feasibility of using OCT to evaluate GI tissue morphology *in situ* and in real-time [171,172]. Since OCT imaging can be performed with small diameter catheters introduced orally or nasally, this emerging technology might be used to screen patients with troublesome upper esophagus symptom for CIP, BE and other changes in the epithelium, in an outpatient clinic without endoscopy or the need for conscious sedation.

### 4.6. Diagnostic Accuracy Using Endoscopic OCT Imaging

Although early detection of Barrett’s metaplasia could decrease morbidity and mortality, the present gold standard to diagnose BE relies upon both endoscopic visualization and histopathologic findings, which are costly and fraught with limited sampling error, as discussed in Section 4.1. The well-accepted Prague classification could guide endoscopic recognition of BE to improve diagnostic yield of biopsies for potential BE [173], but esophagitis which can often be present may confound both endoscopic and histological identification of BE [174,175]. Endoscopic OCT is able to produce high-resolution volumetric imaging of the entire distal esophagus, and thus would have much lower sampling error compared to standard pinch biopsies for BE. Sauk *et al.* performed a clinical study demonstrating that gastroenterologists with limited OCT experience are able to be trained to diagnose intestinal metaplasia simply based on OCT images of the distal esophagus [176]. This study evaluated the inter-observer agreement of 10 readers in diagnosing BE from OCT images. Six of the readers participating in the study including a pathologist had no prior formal training in esophageal OCT image interpretation, while the other readers had extensive experience with OCT technology. All were given a training set and subsequently interpreted a test set. Inter-observer agreement for differentiating mucosa in esophagus *versus* stomach, and BE *versus* non-BE mucosa was determined using multi-rater Fleiss’s κ value which was used to assess the agreement across the readers. Forty-five data sets were included in the test set and all 10 readers had excellent agreement for the OCT differentiation of intestinal metaplasia *versus* non-BE with a κ value of 0.811. For the six readers with limited OCT experience and no formal training, a good agreement was achieved with a κ value of 0.765, while the experienced four readers demonstrated excellent agreement with a κ value of 0.872. They reported that heterogeneous backscatter and irregular surface morphology were the features of BE that most frequently drove the correct diagnosis [169]. This preliminary study concluded that a large group of readers, including both endoscopists and non-endoscopists, would be able to interpret OCT images after being trained and achieve an excellent inter-observer agreement. It is noted that OCT provides full circumferential views of the distal esophagus with micron-level resolution in 2 min, which is much faster than the standard of care requiring endoscopy with biopsies. However, as a potential screening tool, this study did not include pathologic diagnosis as the gold standard in the test due to the lack of registration between the biopsy sites and the OCT imaged sites. Moreover, as a potential surveillance tool, perhaps the more difficult distinction between dysplasia and inflammation or cancer in the setting of BE was not addressed. Therefore, the results reported in this study do not represent the diagnostic accuracy of OCT with respect to histology and will require validation in future histopathologic correlative studies using the laser marking technology mentioned in Section 4.1 [83]. Nonetheless, these preliminary findings suggest that endoscopic OCT can potentially be a promising tool for low-cost screening and surveillance of the distal esophagus.

## 5. Future Applications

Besides the above mentioned clinical research, there are other case studies reporting the use of endoscopic OCT imaging in the GI tract, including ectatic vessels of chronic radiation proctitis (CRP) pre- and post-RFA treatment [88,177], and comparative tissue architectural changes between RFA and CSA [178]. Moreover, several new endoscopic imaging catheter designs have been reported that enhance the visualization of the GI tract [144,146,179,180]. Recently, the first commercialized OCT imaging system specifically for the upper GI tract was developed by NinePoint Medical, Inc. (Cambridge, MA, USA) and has been used in multiple ongoing clinical trials worldwide, also indicating the broad acceptance of endoscopic OCT for clinical applications. Since each imaging technology has its advantages and disadvantages, the development of integrated platforms that combine OCT with other endoscopic imaging modalities can potentially overcome the limitations of individual modalities [138]. For example, endoscopic OCT is capable of visualizing micrometer-scale tissue structural morphologies with intrinsic tissue contrast, and CLE provides sub-micrometer-scale, cellular-level images with endogenous or exogenous contrast. These two complementary imaging modalities can provide important yet different optical information based on unique contrast mechanisms. Moreover, the combination of endoscopic OCT with nanotechnology can potentially introduce new clinical applications including enhanced cancer diagnosis and targeted cancer therapy. The diagnosis and imaging of cancer may be pursued by the intravenous injection and selective accumulation of nanoparticles inside the malignant cells, either passively through the enhanced permeation and retention of solid tumors, or actively in conjunction with antibodies, aptamers, and peptides [181,182]. By generating localized thermal modulation induced by photothermal excitation of the nanoparticles, the phase variation at the targeted regions can be detected using phase-sensitive OCT [183]. The nanoparticles could also be used to induce the apoptosis of the malignant cells by photothermal or photoacoustic microsurgery as well as by the association of cytotoxic drugs [181], which can achieve the targeted cancer treatment. Further improvements in the catheter design and data acquisition technology will allow volumetric imaging with enhanced microscopic resolution and ease of integration with multiple imaging modalities and should enable a wide range of clinical endoscopy applications.

## 6. Conclusions

While endoscopic OCT does not possess the same magnification or contrast as conventional histopathology, it provides unique imaging capability that can visualize tissue microstructure *in vivo* over a large field of view and provides real-time, depth-resolved information with micron scale resolution. With recent advances in swept source and high speed data acquisition technology, ultrahigh speed OCT imaging can be achieved that allows visualization of the tissue dynamic or 3D tissue structure in a short time [146]. In conjunction with standard white light endoscopy, studies have demonstrated the clinical utility of endoscopic OCT for gastroenterology, which includes minimally invasive imaging and optical biopsy without the use of exogenous contrast, volumetric imaging with improved diagnostic yield, and real-time imaging for immediate post-treatment assessment [83,151,155,160,171,172,176,178]. These features make endoscopic OCT particularly suited for detecting small lesions, which can be easily missed if relying on limited random biopsy sampling and on subsequent limited sectioning of the biopsies for pathological inspection. OCT can also discern lesions under the luminal surface. Future efforts are still required to make the most of the endoscopic OCT. With high imaging speed, volumetric OCT data sets with frame-by-frame registration can reveal detailed tissue structure with minimized distortion due to motion or scanning artifacts, so advanced image processing techniques can be used to extract the volumetric information more efficiently. Segmentation techniques can be applied to separate different tissue layers in the squamous mucosa or BE mucosa, and structural features such as thickness of BE epithelium can be automatically quantified immediately after the acquisition of volumetric data set. Pattern recognition techniques can be applied on both cross-sectional and *en face* images to detect other structural features including SSIM, residual/untreated glandular structure immediately after the treatment, and pit patterns of crypts in colon or BE. The development of such techniques will benefit the treatment management by availing structural information immediately to the clinician for appropriate treatment options and dosages to optimize treatment outcomes. Studies of endoscopic OCT for pre- and post-therapy analysis can be conducted with long term follow-up of patients treated with RFA, CSA, endoscopic mucosal resection, photodynamic therapy, argon plasma coagulation, or other emerging endoscopic treatments. Endoscopic OCT could also be studied as an intra-therapy tool for guiding the dosage or resection depth of these therapeutic techniques. Studies in endoscopic OCT screening for dysplasia in the setting of BE or inflammatory bowel disease could also be revisited with improvements in fields of view, resolution, and reliability of 3D information. Long-term clinical studies are needed to decisively establish the clinical utility of endoscopic OCT imaging for broad use beyond the specialty research centers.
